# Effectiveness of Family–Professional Collaboration on Functional Goals Achievement of Children with Cerebral Palsy and Caregivers’ Quality of Life and Burden: A Randomized Comparative Study

**DOI:** 10.3390/jcm13144057

**Published:** 2024-07-11

**Authors:** Sarah L. Al-Otaibi, Maha F. Algabbani, Azza M. Alboraih, Sami S. AlAbdulwahab

**Affiliations:** 1 Department of Health Rehabilitation Sciences, College of Applied Medical Sciences, King Saud University, Riyadh 11433, Saudi Arabiaswahab@ksu.edu.sa (S.S.A.); 2 Research and Scientific Center, Sultan Bin Abdulaziz Humanitarian City, Riyadh 13571, Saudi Arabia

**Keywords:** interprofessional collaboration, parental empowerment, child-centered approach, early intervention, child outcomes, professional support

## Abstract

**Background**: Family–professional collaboration is important to enhance the outcomes for children with cerebral palsy and their caregivers. **Aims**: This study examined the effect of implementing a family–professional collaboration practice model on children with cerebral palsy and their caregivers. **Materials and methods**: A randomized, single-blind comparative study included 28 physical therapists, 44 children with cerebral palsy between the ages of 2 and 12 years old, and their caregivers. Physical therapists in the experimental group received training in how to implement collaboration in their therapy sessions over the course of two sessions (3 h each). The children’s achievement goal-range rate was measured using the Goal Attainment Scaling, the caregivers’ quality of life was assessed using the World Health Organization Quality of Life—Brief, and the caregiver burden was evaluated using the Zarit Burden Interview. **Results**: All children showed improvement on the Goal Attainment Scaling change rate (*p* = 0.002), with no significant differences between groups (*p* < 0.44). However, a group × time interaction was observed. The Children Goal Attainment Scaling rate decreased between the two assessment sessions for children in the control group, while the Goal Attainment Scaling change rate was steady for the experimental group. There were no main effects of time and group or interaction of time × group reported on World Health Organization Quality of Life—Brief domains and no main effect of time on the Zarit Burden Interview, but there was a main effect between groups (*p* = 0.03), with partial eta square = 0.11 in favor of the experimental group. **Conclusions**: The family–professional collaboration practice model could be a potential practice to positively improve the outcomes in children with cerebral palsy and their caregivers.

## 1. Introduction

Family–professional collaboration requires mutual respect and confidence; the sharing of information and skills; open communication; engagement in decision-making; and methods that integrate family beliefs, priorities, and needs into intervention [1,2,3]. It has a positive effect on the children and their families’ outcomes [4].

Family–professional collaboration is identified by two complementary elements: the first element is a relational practice, like displaying respect and empathy and effective listening; the second element is participatory practice, such as family engagement in the intervention procedure by integrating family requirements and preferences into the intervention [1,5,6]. Professionals perform participatory practices less frequently than relational practices, even though participatory practices are more frequently associated with positive results [5,7,8].

The concept of family–professional collaboration provides chances for children to achieve therapeutic goals in association with their activities through everyday routines and is assumed to encourage learning [9,10]. Moreover, collaborative goal-setting and intervention have been found to enhance child development, functional task performance, psychological adjustment, and goal accomplishment [11,12,13,14,15]. The concept of family–professional collaboration also improves caregivers’ emotional well-being and quality of life (QOL), stress levels, burden, satisfaction with healthcare services, sense of competency, and participation in interventions [13,16,17,18,19,20,21].

In 2014, An and Palisano created a family–professional collaboration model to enhance this practice by physical therapists; the family–professional collaboration model encompasses a four-step process to facilitate interaction between family and professionals. Those processes are as follows. (1) Mutually agreed-upon goals: The collaborative process starts with discussing the family’s requirements correlated to child development, preferences, and everyday routines. This discussion results in mutual agreement on intervention goals. (2) Shared planning: The intervention is planned by family and professionals. The family and professional’s mission is agreed upon, and interventions are integrated into the family’s everyday routines. (3) Shared implementation: The family members and professionals collaborate on the implementation of the intervention. While the intervention process is individualized, engaging the family is necessary for the implementation and progression of the intervention plan and for constructing the capability of both family and professionals to address the family’s known needs. (4) Shared evaluation of child and family outcomes: The child and family’s outcomes are evaluated. From a family and professional perspective, individualized outcomes are evaluated. Also, they discuss the changes, successes, and challenges and determine whether or not the goals were accomplished [22]. Moreover, An and Palisano (2017) reported that physical therapists instructed in the four-step process of collaboration interacted in a participatory-practice manner more with the caregivers of children with physical disability compared with other therapists [4]. The model’s effects on children with cerebral palsy (CP) have not been studied exclusively.

The most common physical disability among children is cerebral palsy (CP), which consists of a group of non-progressive disorders that occur in the developing fetal or infant brain and lead to abnormal posture and movement [23,24].

Children with CP often require comprehensive care and support throughout their lives. The collaboration between families and professionals is crucial for providing comprehensive care and support for children with cerebral palsy [25]. By combining their expertise and working together, they can create a holistic and individualized approach to care that optimizes outcomes for the child [4]. Enhanced communication, consistency, empowerment, and continuity of care are among the many benefits of this collaboration [4]. 

Collaboration between the family and professionals can help children and their families set meaningful goals and implement interventions within the family’s conditions, as identified. This is based on their preferences and needs. Many studies have examined the effects of collaborative intervention in nursing and psychology. However, very few studies have examined the impact of collaborative intervention in physical therapy. There were limitations to these studies, including insufficient sample sizes and different pediatric conditions that could have affected the accuracy of results of changes in children’s performance. The majority of the studies included other professional specialties. In general, little research has been conducted to investigate the effects of collaboration on caregivers’ quality of life and burden. No study has been conducted in the physical therapy field on caregivers’ quality of life or burden to our knowledge. Therefore, the objective was to examine the effect of applying the family–professional collaboration model during physical sessions on the (1) functional goals and achievements of children with CP and (2) the caregivers’ quality of life and burden.

This study’s findings may increase physical therapists’ awareness regarding the importance of collaborative intervention processes and their impact on children’s performance and caregivers’ quality of life. The knowledge obtained could also assist organizations in improving their quality-assurance programs and the professional practice of those who treat children with CP in rehabilitation facilities. In addition, it facilitates further research to improve the collaborative intervention process.

## 2. Materials and Methods

### 2.1. Study Design and Setting 

The study was a single-blinded, parallel, randomized comparative study conducted between December 2020 and November 2021. Participants were randomly assigned into two groups: experimental and control group (ratio of 1:1). Physical therapists treating participants in the experimental group received instructional sessions on the family–professional collaboration model’s practice. 

### 2.2. Participants 

Children with CP, accompanying their caregivers and physical therapists, were recruited voluntarily from rehabilitation centers in Riyadh (Sultan Bin Abdulaziz Humanitarian City (SBAHC) and the Children with Disabilities Association (CWDA). Pediatric physical therapists with at least one year’s experience and who are able to communicate in Arabic with children and caregivers were invited to participate by email. In the experimental group, therapists who scored less than 4 out of 5 on their confidence level and who had the ability to implement collaborative strategies were excluded. Children with CP, aged between 2 and 12 years; classified by the Gross Motor Function Classification System (GMFCS) as level I, II, or III; and accompanied by their caregivers were included in the study. Children who had recently undergone surgery (six months) or Botulinum toxin injection treatment (three months), had uncontrolled seizures, or had discontinued physical therapy sessions were excluded.

### 2.3. Sample Size Calculation

The sample size was computed using the *Power Sample Size and Calculation Program* (version 3.1.6) to find a large effect size (d = 0.8) based on a previous study report [4], a significance of 0.05 (one-tail), and a power level of 0.80, with 42 pairs of participants (children with CP and their caregivers). For the possibility of drop-off, 20% of the estimated sample were added (8 pairs).

According to the literature, each physical therapist treated 1 or 2 children as a maximum ratio [4]. Therefore, at least 26 physical therapists were required.

### 2.4. Ethical Considerations and Consent 

Ethics approval was obtained from the Institutional Review Board (IRB) of the College of Medicine at King Saud University (KSU) (No. E-20-4777) and SBAHC (No. 36-2020-IRB). Permission from CWDA was attained. Physical therapists and caregivers signed an informed-consent form before the study started. Essent was obtained from the children, as appropriate. Furthermore, permission was gained to use the Arabic version of the Zarit Burden Interview (ZBI) from the publisher [26]. This study was retrospective and registered at clinicaltrials.gov, with the ID No. NCT05709080.

### 2.5. Sampling Method, Randomization and Blindness

Children and physical therapists were randomly selected from eligible participants using a computer random-number generator. Second, a computer random-number generator randomly assigned physical therapists to experimental or control groups. In addition, a simple coin-flipping method was used to randomize children. Each pair of children was considered together to ensure an unbiased allocation of participants. The coin-toss outcome determined which child would be assigned to the experimental group and which would be assigned to the control group—no more than two children for each therapist. The details of the study were concealed from the in-charge therapists. Participants were blinded and unaware of the intervention groups.

### 2.6. Instructional Sessions

The principal investigator trained physical therapists in the experimental group on how to conduct a collaborative intervention. This was achieved by following the process and strategies of the family–professional collaboration practice model to enhance collaboration during physical therapy sessions [22]. They were asked to rate their confidence level to implement the collaboration process twice (one week after the first instructional session and before the start of intervention) using a five-point Likert scale (5 = greatly confident, 4 = fairly confident, 3 = moderately confident 2 = somewhat confident, and 1 = not at all confident). Any step that therapists indicated as difficult or that they were not confidant to apply was reviewed. According to the confidence-level score, all therapists met the inclusion criteria. Therapists were advised not to share any of the instructions with the therapist in the control group.

The instructions were conducted online in two sessions for six hours (three hours per session) for small groups (six therapists per each group) [4]. The family–professional collaboration (four-step process) model was explained in the first instructional session as follows. Step 1: Mutually agreed-upon goals—Therapists were instructed to involve the family in goal-setting by facilitating conversation to (1) get to know a child’s interests, previous experiences, challenges, and family preferences and priorities; and (2) to enable the family to orient toward positive changes in the immediate future and to determine what will be changed when the intervention plan is successful. This conversation is based on the client-centered interview process used for the Canadian Occupational Performance Measure (COPM) [22]. Step 2: Shared planning—Therapists were instructed to develop an intervention plan that meets the child and family’s needs. The therapist facilitates shared planning and integrates intervention into the family’s daily routines and activities. Step 3: Shared implementation—The therapist is instructed to continuously work with the caregivers while applying the intervention and adjust the intervention plan if needed. Step 4: Shared evaluation of child and family’s outcomes—Therapists were instructed to involve the caregivers to determine whether the intervention was effective or not and if the goals were reached. By the end of this session, additional information and a summary of the instructions were sent to the therapist.

The second instructional session was conducted within 2 weeks from the first session. At the beginning of this session, the confidence level to implement the collaborations process was discussed. Therapists were instructed to practice the steps with each other, under the researcher supervision, following the strategies and examples of questions listed in An and Palisano’s study to promote parent engagement in the intervention [22].

The principal investigator attended at least one practice session with each therapist to ensure that he or she practiced the collaborative model correctly. Furthermore, the principal investigator met regularly with the therapists after each treatment session during the intervention.

Physical therapists in the control group met with the researcher once through an online meeting. They did not receive any instructions related to the collaborative intervention process. The meeting was limited to instructing the therapist to conduct the therapy sessions to achieve the functional goals as usual. Physical therapists in the control group have used the routine intervention process.

All therapist were asked to set one or two intervention goals for children that related to everyday or leisure activities at home and community based on the two domains of the International Classification of Functioning, Disability, and Health for Child and Youth version (ICF-CY): activity limited and participation restricted [27].

### 2.7. Outcome Measures

#### 2.7.1. Goals Attainments Scale

The Goals Attainment Scale (GAS) is a standardized scoring procedure designed to evaluate goal achievement and quantify meaningful change. The GAS is composed of a Likert scale of 5 points, where the lowest scores are (−2) at baseline and (−1) mean progression toward the goals but have not reached them; (0) represents the expected level after the intervention, (+1) indicates a better level than expected, and (+2) indicates the highest possible level [28]. Clinically, GAS can be used to compare goal achievement across groups and individuals [29]. GAS showed good validity and reliability [30,31]. Furthermore, it displayed excellent sensitivity to change, as has been verified in different contexts and populations [32,33,34,35,36]. A major advantage of GAS over COPM is that it allows for more flexibility in setting goals. This property makes GAS an appropriate outcome measure for rehabilitation programs and studies whose objectives relate to family-directed goals. GAS may also be helpful if the study requires unique goals that can be difficult to assess with a standardized tool. GAS is analyzed by transforming the goal-achievement rating to the T-Score, a standardized measure with a mean of (50) and a standard deviation of (10). Consequently, if the goals were met, the T-score was 50; exceeding goal expectations resulted in a T-score higher than 50, and failing to achieve goals resulted in a T-score of less than 50 [37,38]. In This study, the outcome scores of the goals were converted using a computerized program that calculates the baseline score, the T-Score (achieved score), and the change score through an available spreadsheet calculator [39].

#### 2.7.2. World Health Organization Quality of Life—Brief

The Arabic version of the World Health Organization Quality of Life—Brief version (WHOQOL-BREF) was used to assess the QOL of the caregivers. It is a self-report questionnaire focused on measuring the respondents’ perception of the last two weeks before administration [40,41]. The four domains of QOL that are identified by WHO were assessed as follows: physical health (7 items), psychological health (6 items), social relationships (3 items), and environment (8 items). These items were scored on a five-point Likert scale, ranging from (1) mean strongly agree to (5) mean strongly disagree; the overall QOL calculated by responses mean scores of each subscale [42,43]. Using raw scores on the four domains, a 4–20 scale was transformed linearly into a 0–100 scale, where 100 is the highest and 0 is the lowest. The higher score indicates a better quality of life [44]. Previous studies identified that the cutoff point was ≥60 points for a good level of QoL [45]. The Arabic version showed good validity (factor analysis range from 0.53 to 0.66), the test–retest reliability with a 95% confidence interval was 0.95 (0.94–0.97), and the internal consistency using Cronbach’s Alpha was 0.9 [46].

#### 2.7.3. Zarit Burden Interview—Short Version

To measure caregiver burden, the Arabic Zarit Burden Interview—short version (ZBI-A) was used [26,47]. A 5-point Likert scale is used to rate 12 items, ranging from (0), which represents almost never, to (4), which represents almost always. Scores ranged from 0 to 48, with a higher score indicating greater burden [26]. A score of 17 or more was known as a high burden level [48]. The ZBI is valid and reliable (Cronbach’s Alpha = 0.77) [26].

### 2.8. Procedure

#### 2.8.1. Data Collection Process

The caregiver completed the demographic sheet information related to their children or themselves prior to the first session. The GAS change rate was measured twice, in the third week and the last week. The caregiver completed the WHOQOL-BREF and ZBI questionnaires twice, in the first and last sessions.

#### 2.8.2. Intervention

The children received intensive physical therapy sessions for six weeks (5 sessions/week), and each session lasted 45–60 min. During the first sessions, the assessments were completed, and the intervention goals were determined. In the second session, planning the intervention and starting the treatment. The re-assessment session was conducted on the third week, and the final assessment was conducted in the last week.

### 2.9. Data Analysis

Data analyses were processed using IBM SPSS Statistics for Windows, version 28 (IBM Corp., Armonk, NY, USA). The Shapiro–Wilk test was utilized to examine the normality of the data for continuous variables. The descriptive statistics were presented as mean and standard deviations if the data were normally distributed or as median and (1st–3rd quartiles) if the data were non-normally distributed. Frequency and percentage were used to present the categorical data. The comparison between the experimental and control groups at the baseline level was computed using the independent sample *t*-test for normally distributed data, the Mann–Whitney test for non-normally distributed data, and the chi-square or Fisher exact test (if the number of observations was less than 5) for categorical data. Mixed analysis of variance (ANOVA) was used to analyze the effect of physical therapists’ training on the collaborative model on the group (experimental and control), time (first assessment and last assessment), and group-by-time interaction for all dependent variables (GAS changed rate, WHOQOL-BREF domains, and ZBI). Levene’s test was used to indicate homogeneity of variances between the groups, and equal covariances were determined according to Box’s M test (*p* > 0.05). The effect size was interpreted depending on the guidelines proposed by Cohen (1988). The effect size was considered as follows: <0.1 = trivial effect, 0.1–0.3 = small effect, 0.3–0.5 = moderate effect, and >0.5 = large difference effect with 95% confidence interval [49].

## 3. Results

**Figure 1:**
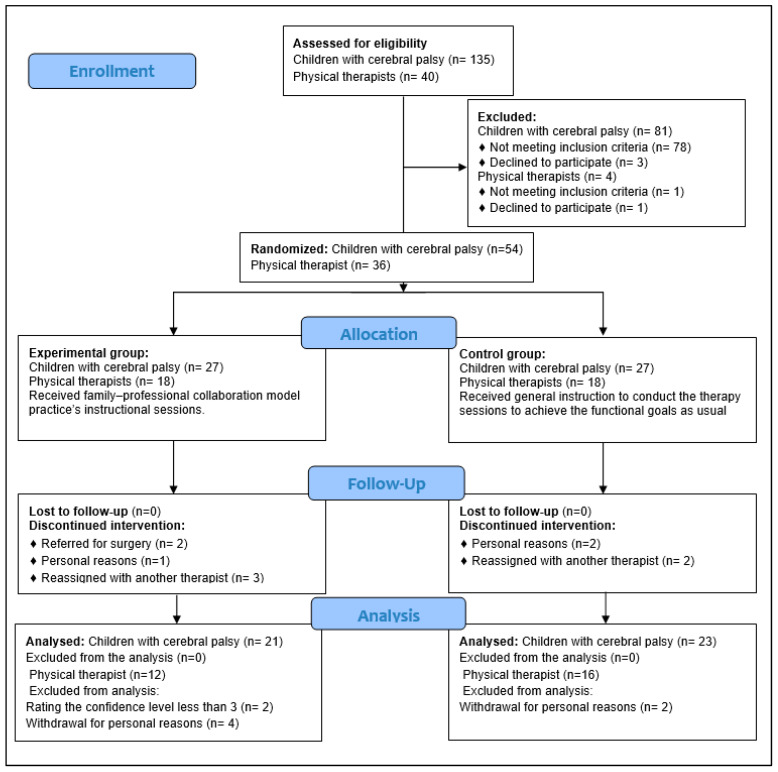
Participants’ flow diagram.

The Shapiro–Wilk test showed that the data were normally distributed (*p* > 0.05), except for children’s age and physical therapists’ experience (*p* < 0.05). This study recruited 32 physical therapists and 54 pairs of children and their caregivers. Four physical therapists withdrew from the experimental group due to personal reasons. Six children were excluded (four children whose therapists withdrew from the study, one child who was referred to surgery, and one child who discontinued sessions). In the control group, four children discontinued the physical therapy session due to personal reasons. The final data analysis included 44 pairs (21 in the experimental group and 23 in the control group) and 28 physical therapists (12 in the experimental group and 16 in the control group; Figure 1).

### 3.1. Participants’ Characteristics

The median of physical therapists’ experience in pediatric physical therapy was 4.5 (3–16.5) years. Out of the 28 physical therapists, 20 were females. At baseline, the Mann–Whitney test indicated no significant differences between physical therapists’ experience in the experimental group median of 5.00 (2.25–17.75) and in the control group median of 4.00 (3–13.75) (U = 84, *p* = 0.59).

Children and caregivers’ characteristics are presented in Table 1. The children with CP were aged between 2 and 12 years old, with a median of 6 (3.25–8.75 years). All children were accompanied by their caregivers, with mothers representing 34 (77.3%) and other relatives representing 10 (22.7%). There were no significant differences between the children and caregivers’ demographic and clinical characteristics at baseline (*p* > 0.05). However, the levels of caregivers’ burden were significantly higher in the experimental group at baseline (Table 1).

### 3.2. Effect of Family–Professional Collaboration on Children’s Performance

**Figure 2:**
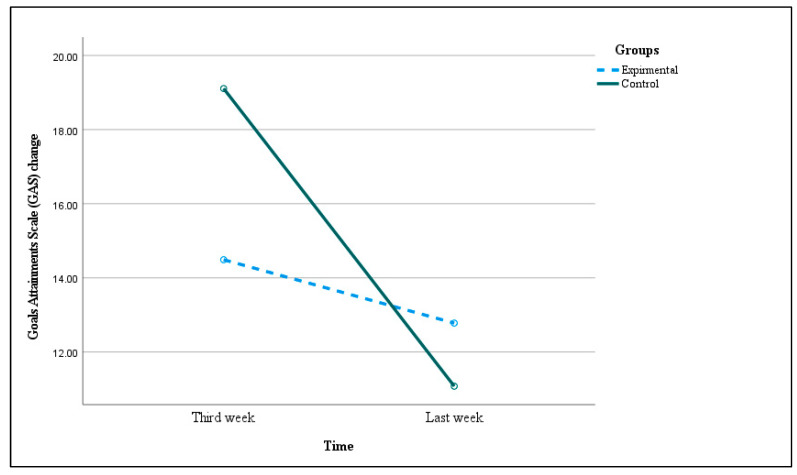
ANOVA test of the interaction between time and group for GAS (first assessment and last assessment), F (1, 42) = 4.53, and *p* = 0.04, and ηp^2^ = 0.10. There were 21 children in the experimental group and 23 children in the control group.

The ANOVA showed a main effect of time on the GAS changed rate (F (1, 42) = 10.76, *p* = 0.002, and ηp^2^ = 0.20) and no main effect of group (F (1, 42) = 0.94, *p* < 0.44, and ηp^2^ = 0.02). Both groups showed improvement in GAS change rate, with no significant differences between them. However, an interaction between time and group was presented on the GAS changed rate (F (1, 42) = 4.53, *p* = 0.04, and ηp^2^ = 0.10; small effect; Figure 2). Most of the improvements for the control group happened during the first re-assessment session (third week of physical therapy intervention). The children in the control group showed a higher GAS change rate during the first re-assessment (19.11, SE 1.48), and the rate of change decreased during the last assessment (11.08, SE 1.44). While the experimental group revealed no significant differences in the rate of GAS changes between the first re-assessment and the last assessment (14.49, SE 1.55; 12.78, SE 1.5, respectively). The experimental group showed greater changes than the control group in the last assessment, though the change did not reach the significance level.

### 3.3. Effect Family–Professional Collaboration on Caregivers’ Quality of Life and Burden

**Figure 3:**
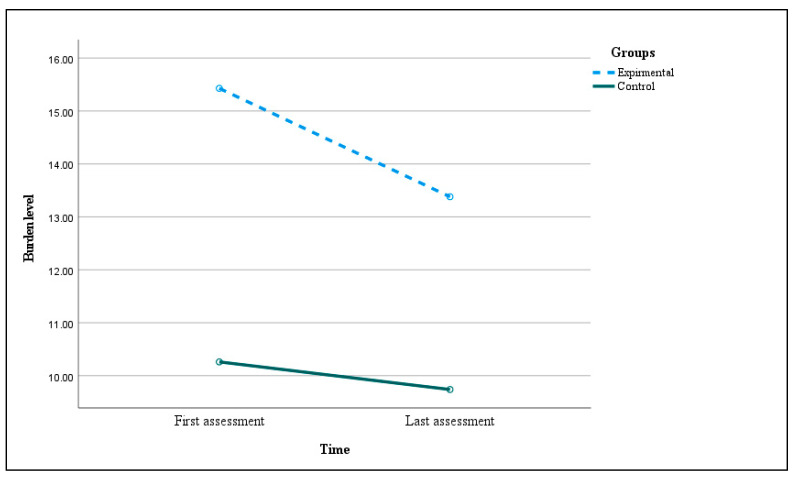
ANOVA test of the interaction between time and group for ZBI (first assessment and last assessment), F (1, 42) = 5.06, *p* = 0.03, and ηp^2^ = 0.11. There were 21 children in the experimental group and 23 children in the control group. Interaction between time × group for ZBI (first assessment and last assessment).

There were no main effects or interactions of time or group reported on quality-of-life domains (Table 2). Furthermore, there was no main effect of time on Burden F (1, 42) = 0.87, *p* = 0.36, ηp^2^ = 0.02 but there was a main effect of groups F (1, 42) = 5.06, *p* = 0.03, ηp^2^ = 0.11. The burden was decreased for the experimental group from 15.43 ± 1.56 (SE) in the first assessment to 13.38 ± 1.51 (SE) in the last assessment. The control group had a slightly lower result in the last assessment (9.74, SE 1.44) rather than the first assessment (10.26, SE 1.49). Although there was a mean difference between the experimental and control at baseline (mean differences 4.62 SE 2.14 *p* = 0.04) in the favored to control group, the differences decreased after the intervention (mean differences 1.70 SE 2.09, *p* = 0.42) (Figure 3), the effect size for experimental was moderate effect (0.31) and for control was small effect (0.12). There was no interaction, F (1, 42) = 2.46, *p* = 0.12, ηp^2^ = 0.06.

## 4. Discussion

This study examined the impact of applying the family–professional collaboration (four-step) practice model during physical sessions on the performance of children with CP toward attaining their goals and on the caregiver’s QOL and burden.

Children in both the experiment and control groups improved in their performance toward their intervention goals, according to this study. They did not differ significantly. Although the experimental group showed slightly greater changes in GAS than the control group, this did not reach significance. In addition, the QOL domains for caregivers did not show statistically significant levels within each group and between the two groups. In contrast, the burden on caregivers was significantly different between groups, favoring the experimental group. These results were consistent with An et al.’s study (2017) [4], which applied the same four collaborative strategies as the current study. The researchers found that the children’s performance increased in the experimental and control groups (*p* < 0.05), but there was no difference between the groups, and the effect size of children’s performance, which changed scores, was the medium effect (0.73), which was in favor of the experimental group when the achievement was measured by the COMP.

In this trial, the experimental and control groups showed an increase in the GAS change rate after the first three weeks of intervention. The GAS change rate remained steady in the experimental group. In the control group, the rate of change decreased with no significant differences at the end of the intervention. The rate of achieving the functional goals was similar to that of a study by Imms et al. in 2018; they found that a higher change rate to achieve the functional goals selected by the child and family for children with CP occurred during the first four weeks of intervention compared to eight weeks, measured by the COPM and GAS, without any differences between the two groups at eight weeks, and even though the results did not reach the significance level [50].

The reasons for the variation are unclear but could be attributed to the motivation in the children and their families being better after continuous engagement of the therapy sessions by mutually agreeing on the interventional goals, discussing the treatment plan, determining the roles during the intervention, and motivating the caregivers to share their observations and adjust the interventional plan accordingly. Consequently, it resulted in maintaining the improvement for children in the experimental group throughout the physical therapy intervention period. The supportive evidence indicated that involving the family in goal-setting and measuring the change may have benefits for their perception of the performance of their child [10,51,52].

Children with GMFCS levels I to III enrolled in this study to eliminate any differences in the performance change rate. The previous study indicated that GMFCS levels were not statistically significant in the group with high COPM change scores and were not associated with change scores. However, the sample size for each level was very small in detecting the differences, and the GMFCS level was considered clinically important in the achievement of the goals [52]. In addition, the age of the children and their GMFCS level were not statistically significant between the groups at the beginning of the current study.

The intervention goals in the present study were a simple goal unified to address the activities’ limitation and participation restriction domains of ICF (no more than two functional activities). Also, using the GAS change rate to measure the score of change for all goals helped eliminate the individual differences in achievement scores for each child’s identified goals. The authors of a previous randomized trial found minor differences in some goal characteristics, which do not serve to conclude that the nature of the intervention goals for children with CP could explain differences in children’s performance levels. They had a combination of simple and complex goals over various ICF domains [52].

The four domains of the QoL for caregivers in the experimental and control group did not improve after intervention. However, the scores were above 60 points, indicating a good QoL. The levels of QoL were consist with a study conducted by Adegoke and Akosile in 2014 [53]. No improvement of the QoL could be seen as a result of the short duration between the assessment sessions (6 weeks) that led to no significant changes on the QoL. Moreover, the scores of each domain at baseline were good (all domain scores were over 60 points). To the authors’ knowledge, the effect of the collaborative intervention on the caregivers’ QOL has not been addressed before in the physical therapy field, and this distinguishes the present study.

Additionally, the intervention did not have a significant effect on caregivers’ burdens measured by ZBI between the first and last assessment. However, the burden scores were lower than the cutoff point (17). As a result of the convenient sampling method and random assignment to either group, there was no control over the burden level at baseline. The burden level differs significantly at baseline, where the caregiver burden in the experimental group was higher. However, there was a positive main effect between groups in the last assessment of the ZBI that favored the experimental group. In agreement with our observation, Haghgoo et al.’s study (2018) showed that the level of burden for the caregivers of patients with mental disorders was reduced significantly in the experimental group, which received the collaborative care model after 11 sessions of therapy, compared to caregivers in the control group, who received the usual intervention [54].

Furthermore, other factors have shown to be associated with QoL and burden level, such as education level. Several studies have consistently shown that individuals with higher levels of education tend to experience better QoL [55,56,57]. This is likely because education provides individuals with knowledge, skills, and cognitive abilities that enable them to navigate various aspects of life more effectively. Educated individuals tend to have a better understanding of their health conditions, treatment options, and coping mechanisms, which can lead to improved outcomes.

In addition, working status significantly impacts QoL and burden, as employed individuals are more likely to report higher QoL levels than unemployed individuals [55,58]. It may be because employment provides financial security, social support, meaning, and purpose. Additionally, job satisfaction and financial stability can improve QoL and reduce burdens. In this study, there were no significant differences between these factors at baseline.

## 5. Limitations and Recommendations

The children and their caregivers during the sessions: Children with CP (GMFCS levels IV and V) were not included in the study due to differences in progression, severity, and associated impairments that could affect management. Consequently, the results of this study cannot be generalized. In the first assessment, the experimental group had a higher burden level than the control group. The study evaluated only the short-term effect of the intervention. We recommend conducting studies with a double-blind design, with a larger and matched sample, including (GMFCS levels IV and V). Moreover, the study considered the effect of collaboration in different conditions and rehabilitation settings. We recommend considering the assessment of anxiety and depression of the caregivers.

## 6. Conclusions

This study showed that the means of GAS rating changes increased after the intervention for the children but did not reach the significance level between the experimental and control groups. Nevertheless, the experimental group’s gas changing rate continued at the same range during the intervention session, contrary to the control group, whose GAS change decreased after the first sessions.

Furthermore, the caregivers’ QOL showed no significant improvement. The burden decreased significantly for the experimental group compared to the control group. Finally, these results may raise physical therapists’ awareness about the importance of delivering collaborative intervention and provide insight for further research.

## Figures and Tables

**Table 1:** Demographic and clinical characteristics of the participant.

Characteristics	Experimental Group N = 21	Control Group N = 23	Comparison at Baseline
Children			
Age (years)	6 (4–8)	6 (3–9)	U = 23, *p* = 0.73
Gender:			
Boys	16 (76.2%)	11 (47.8%)	X^2^ = 3.72, *p* = 0.05
Girls	5 (23.8%)	12 (52.2%)	
GMFCS levels:			
Classification (I)	0 (0%)	1 (4.3%)	Fisher exact test, *p* = 0.49
Classification (II)	6 (28.6%)	5 (21.7%)	
Classification (III)	15 (71.4%)	17 (73.9%)	
Caregivers			
Number	21	23	-
Age (years)	35.90 ± 7.20	34.04 ± 6.78	t(42) = 0.06, *p* = 0.38
Caregivers’ Education:			
Secondary or lower	7 (33.4%)	10 (43.5)	X^2^ = 8.30, *p* = 0.30
Bachelor’s Degree or higher	14 (66.6)	13 (56.5%)	
Working status:			
Full time	10 (50%)	6 (27.3%)	X^2^ = 2.29, *p* = 0.13
House	10 (50%)	16 (72.7%)	
Financial level:			
<3000	1 (4.8%)	2 (8.7%)	Fisher exact test, *p* = 0.98
3000–6000	6 (28.6%)	6 (26.1%)	
6001–9000	5 (23.8%)	7 (30.4%)	
9001–12,000	4 (19.0%)	4 (17.4%)	
>12,000	5 (23.8%)	4 (17.4%)	
QOLWHO-BREF domains:			
Physical health	65.90 ± 16.720	70.83 ± 16.322	t(42) = −0.77, *p* = 0.45
Psychological health	65.00 ± 17.544	66.61 ± 17.466	t(42) = −0.16, *p* = 0.87
Social relationships	67.75 ± 19.177	79.35 ± 19.915	t(42) = −1.87, *p* = 0.07
Environment	62.65 ± 17.098	67.57 ± 12.591	t(42) = −0.89, *p* = 0.38
ZBI	16.20 ± 7.250	10.26 ± 6.380	t(42) = 2.39, *p* = 0.02 *

Data are represented as mean ± SD unless otherwise stated. Data represented as median (1st–3rd quartiles) for children’s age, frequency, and percentage (for gender; GMFCS; and caregiver’s education, working status, and financial level). % = percent; U = Mann–Whitney; X^2^ = chi-square; t = independent simple *t*-test; GMFCS = Gross Motor Function Classification System; QOLWHO-BREF = World Health Organization Quality of Life—Brief; ZBI = Zierat Burden interview. * Significant difference *p* < 0.05.

**Table 2:** Result of the effect of the family–professional collaboration on caregivers’ Quality of life (WHOQOL-BREF) (within-subjects effect, between-subjects effect, and time–group interaction).

	Within-Subjects Effect			Between-Subjects Effect			Interaction		
WHOQOL-BREF Domains:	F	*p*	ηp^2^	F	*P*	ηp^2^	F	*p*	ηp^2^
Physical health	0.559	0.459	0.013	0.606	0.441	0.014	0.019	0.892	0.000
Psychological health	1.778	0.190	0.041	0.455	0.504	0.011	0.635	0.430	0.015
Social relationships	0.000	0.999	0.000	3.071	0.087	0.068	1.089	0.303	0.025
Environmental	0.007	0.344	0.021	0.965	0.331	0.022	0.007	0.934	0.000

F = magnitude of effect; ηp^2^ = partial eta square (effect size). Significance level < 0.05.

## Data Availability

The data that support the findings of this study are available upon request from the corresponding author. The data are not publicly available due to their containing information that could compromise the privacy of research participants.
